# *Nannizziopsis pluriseptata*-Associated Skin Lesions in a Shingleback Skink (*Tiliqua rugosa*) and Spiny-Tailed Skinks (*Egernia depressa*) in Australia

**DOI:** 10.3390/vetsci13020162

**Published:** 2026-02-06

**Authors:** Victor A. Palma Jauregui, Stephanie Shaw, Jana Schader, Richelle G. Butcher, Rachael Clark, Timothy H. Hyndman, Viviana Gonzalez-Astudillo

**Affiliations:** 1 Facultad de Medicina Veterinaria y Zootecnia, Universidad Nacional Autónoma de México, Mexico City 04510, Mexico; 2 Moggill Koala Rehabilitation Centre, Queensland Department of the Environment, Tourism, Science and Innovation, Moggill, QLD 4070, Australia; 3 Avifauna Vet, Bulli, NSW 2516, Australia; 4 Biodiversity Health Research Team, School of the Environment, The University of Queensland, St Lucia, QLD 4072, Australia; 5 School of Veterinary Science, The University of Queensland, Gatton, QLD 4343, Australia; 6 School of Veterinary Medicine, Murdoch University, Murdoch, WA 6150, Australia

**Keywords:** *Nannizziopsis pluriseptata*, shingleback skinks, spiny-tailed skinks, Australia

## Abstract

Both captive and free-living reptiles are susceptible to infection by fungi from the *Nannizziopsis* genus and are prone to developing severe lesions that can affect skin and deeper tissues, becoming multi-systemic. With Australia having close to 1000 reptile species—of which 93% are endemic—the prevalence, distribution and susceptibility of reptiles to this emerging fungal infection remain unknown. In this case series, captive skinks originating from the states of Queensland and Western Australia were presented to wildlife veterinarians with dermatitis. *Nannizziopsis pluriseptata* was identified via fungal isolation, PCR (polymerase chain reaction) and sequencing, confirming the first record of this pathogen in the country.

## Simple Summary

Emerging fungal diseases are a growing concern for the health of free-living reptile populations. From these, *Nannizziopsis* species are noted as important pathogens associated with both skin and systemic disease. This case series describes skin lesions associated with *Nannizziopsis pluriseptata*, in captive native Australian skink species of unknown origin from two different states. It also provides a review on the clinical, pathological and diagnostic features of this emerging pathogen, currently of unknown impact to Australian herpetofauna.

## 1. Introduction

Fungi are considered important emerging pathogens for several animal species, causing severe diseases such as chytridiomycosis caused by *Batrachochytrium dendrobatidis* and *B. salamandrivorans* in amphibians, or white-nose syndrome in bats, associated with *Pseudogymnoascus destructans* infection [1,2]. Fungal infections have led to wildlife population declines, species extinctions and even disturbances in macroecological patterns, as they represent a great risk for numerous vertebrate species [1,2]. Several fungal diseases have been documented in numerous reptile species as well [3,4,5].

The most important emerging fungal pathogens of reptiles belong to the order Onygenales, primarily *Nannizziopsis* spp., *Paranannizziopsis* spp., and *Ophidiomyces ophidiicola* (family *Onygenaceae*) [6]. From these, the genus *Nannizziopsis* has gained notoriety since the late 1990s and is of particular concern for Australia due to its broader host distribution, spillover potential to native herpetofauna, and clinical severity. The *Nannizziopsis* genus is constituted of 12 species that are known to infect vertebrates, of which *N. vriesii*, *N. barbatae*, *N. dermatitidis, N. chlamydospora*, *N. draconii*, *N. arthrosporioides*, *N. pluriseptata*, *N. guarroi* and *N. crocodili* are known to infect reptiles, while, *N. infrequens, N. hominis* and *N. obscura* have only been found in human patients [7]. The typical manifestation of *Nannizziopsis* spp. infection involves a dermatitis characterized by epidermal proliferation, erythema, crusting, necrosis, and/or ulceration anywhere on the body but often affecting ventral skin [8,9]. Dermal infections can extend deeper and cause muscle and bone necrosis, and even multi-systemic disease with granulomas in the nasal cavity, cornea, conjunctiva, heart, liver and lungs, compromising the host and resulting in severe emaciation [9,10,11,12,13,14]. Most of the *Nannizziopsis* species currently known were previously described as part of the *Chrysosporium* anamorph of *Nannizziopsis vriesii* (CANV) complex due to the morphological similarities between them and the lack of molecular characterization of the isolates [7]. Infections by CANV complex fungi were formerly colloquially known as “yellow fungus disease” due to the characteristic gross skin lesions; however, the term “nannizziomycosis” is currently preferred [8].

Australia is home to approximately 10% of all *Squamata* species in the world, the largest diversity of any country, and the Australian state of Queensland is habitat to some of the most imperiled species of snakes and lizards [15]. In addition, Australia hosts the largest species diversity of skinks in the world with more than 500 species [16]. Thus, the incursion or recent discovery of any fungal pathogen, especially of notably lethal ones such as *Nannizziopsis* spp., holds high conservation relevance. Currently, the most frequently isolated *Nannizziopsis* species in Queensland is *Nannizziopsis barbatae*, a species that has caused at least one major outbreak in dozens of eastern water dragons (*Intellagama lesueurii lesueurii*) in the state, with other cases in a tommy roundhead dragon (*Diporiphora australis)*, an eastern blue tongue skink (*Tiliqua scincoides scincoides*), a shingleback skink (*Tiliqua rugosa*) and a centralian blue tongue skink (*Tiliqua multifasciata*) from across the country [9,17].

The following study describes *Nannizziopsis pluriseptata* infection in two Australian skink species; the first report since this pathogen was identified from the skin of a five-lined skink (*Plestiodon inexpectatus*) in North America [4]. The history of the skinks in the present report is unknown, except for the fact that they were confiscated from illegal trade from regions in Australia that are more than 4200 kms apart.

## 2. Materials and Methods

### 2.1. Population

Four shingleback skinks (*Tiliqua rugosa*) were intercepted in the mail by authorities in the state of Queensland by the Department of Environment, Tourism, Science and Innovation (DETSI). The skinks were held in captivity for an unknown period and intended for illegal export. Subsequently, they were submitted to the state wildlife veterinarian for assessment. All individuals had crusty skin lesions and two were subsequently euthanized due to dermatitis and stomatitis. Two individuals were submitted for postmortem examination in August 2022 (Cases 1 and 2). Sections of affected skin from these two skinks were submitted for PCR testing for *Nannizziopsis* spp.

Forty pygmy spiny-tailed skinks (*Egernia depressa*) allegedly wild caught in the state of Western Australia, were held in two consignments. These were subsequently detected and seized by federal officers from the Department of Climate Change, Energy, the Environment and Water (DCCEEW) at a post office under section 303DD of the Environment Protection and Biodiversity Conservation Act 1999 (EPBC Act) before they were set to be shipped overseas. These skinks were transported in poorly ventilated containers and most showed skin lesions and emaciation. One individual was deceased, and two were euthanized by a veterinarian due to concerns over the presence of skin lesions. Two of these three skinks were submitted for postmortem examination and organ samples were sent for histological assessment in October 2023 (Cases 3 and 4). Skin swabs collected from these two skinks were submitted for PCR testing for *Nannizziopsis* spp.

### 2.2. Histological Assessment

Tissues collected during postmortem examinations were fixed in a 10% neutral buffered formalin solution and processed by standard histology procedures, embedded in paraffin, sectioned at 5 µm and stained with hematoxylin and eosin (H&E), Grocott’s methenamine silver (GMS) and Periodic acid–Schiff (PAS). Sections of formalin-fixed paraffin-embedded tissue from Case 1 were submitted for PCR testing for *Nannizziopsis* spp.

### 2.3. Fungal Culture and Identification

Fungal isolation was conducted on skin biopsies from Cases 1 and 2, by placing a ~5 × 5 mm segment of cutaneous lesion directly onto Sabouraud Dextrose Agar containing Chloramphenicol (SDA) (Oxoid, Thermo Fisher, Basingstoke, UK) and Potato Dextrose Agar (PDA) (Oxoid, Thermo Fisher, Basingstoke, UK). Plates were incubated at 30 °C for 21–28 days. Suspect colonies were subcultured onto PDA, following 21 days of incubation. Morphological features were examined using lactophenol blue solution (Sigma-Aldrich, Merck, Darmstadt, Germany) under light microscopy. Isolates with morphological features consistent with *Nannizziopsis* spp. were submitted to the Australian National Mycology Reference Laboratory for confirmation by 18S PCR.

### 2.4. DNA Extraction, PCR and Sanger Sequencing

DNA (deoxyribonucleic acid) extraction and PCR targeting a fragment of the rRNA-ITS (ribosomal ribonucleic acid-internal transcribed spacer) region were conducted using cutaneous lesion samples. Genomic DNA was extracted from skin swabs using the Purelink Viral RNA/DNA Mini Kit (Thermo Fisher, Waltham, MA, USA) according to the manufacturer’s instructions. Swabs were vortexed (~30 s) in sterile saline (~1 mL) before a 200 µL aliquot was added to carrier RNA, a lysis buffer and proteinase K, and then processed as per the manufacturer’s instructions. The Indispin Pathogen Kit (Indical Bioscience, Leipzig, Germany) was used to extract nucleic acid from fresh frozen skin. Thawed tissue segments of ~ 3 × 3 × 3 mm were first homogenized in 400 µL of PCR-grade water and 200 µL of phenol (pH 8, Sigma, St. Louis, MI, USA) using a Mini-Beadbeater 24 (Biospec, Bartlesville, OK, USA) at 3000 oscillations per minute for 2 min using 0.5 mL of silicone-carbide sharp particles (1 mm diameter; Biospec, Bartlesville, OK, USA). Following homogenization, the manufacturer’s extractions were followed. Formalin-fixed paraffin-embedded (FFPE) tissues were treated the same as fresh tissues except that FFPE tissues were first deparaffinized in two washes of xylene and two washes of 100% ethanol. The deparaffinized FFPE samples were then incubated overnight in proteinase K at 56 °C, and then at 90 °C for six hours [18]. All nucleic acid extractions were eluted into 30 µL of PCR-grade water.

PCR testing was conducted targeting a 261 nucleotide DNA fragment of the rRNA-ITS gene cluster of *Nannizziopsis* species, which was amplified using 10 µL of 2× master mix from the PlatinumTM Green Hot Start PCR Master Mix (2X) (Thermo Fisher, Waltham, MA, USA), forward 5′GCA TCG ATG AAG AAC GCA GCGA and reverse 5′GGY CAG CKCCG GCC GGGTC primers at 500 nM final concentration, and 1 µL of extracted nucleic acid, with the final volume brought to 20 µL using PCR-grade water. Thermal cycling conditions were as follows: 94 °C for 2 min, followed by 10 cycles of 94 °C for 20 s, 72 °C for 45 s (decreased by 1 °C per cycle), 72 °C for 30 s, followed by 30 cycles of 94 °C for 20 s, 62 °C for 45 s, 72 °C for 30 s. A no template extraction was included as a negative control. Following gel electrophoresis, amplicons near in size to 261 bp were cut from the gel for Sanger sequencing using standard methods [9].

After primer sequences were edited out, the *Nannizziopsis* sequences were used as queries to search the NCBI (National Center for Biotechnology Information) non-redundant nucleotide database using BLASTN (Basic Local Alignment Search Tool Nucleotide version 2.17.0).

### 2.5. Phylogeny

Nucleotide sequences of the cognate region of the rRNA-ITS2 cluster were retrieved from GenBank for 12 *Nannizziopsis* spp. and *Paranannizziopsis australasiensis*. Sequences were aligned using MUSCLE (Multiple Sequence Comparison by Log-Expectation) [19]. To ensure homologous comparison, nucleotides outside the region corresponding to the skink nannizziopsid sequences were removed. The best-fit nucleotide substitution model was identified in MEGA version 11 using the Bayesian information criterion [20]. Phylogenetic relationships were inferred using the Maximum Likelihood method with the Tamura 3-parameter (T92) model [21]. The partial deletion option was applied to eliminate all positions with less than 95% site coverage. Node support was evaluated using 1000 bootstrap replications.

## 3. Results

### 3.1. Gross Lesions

**Figure 1:**
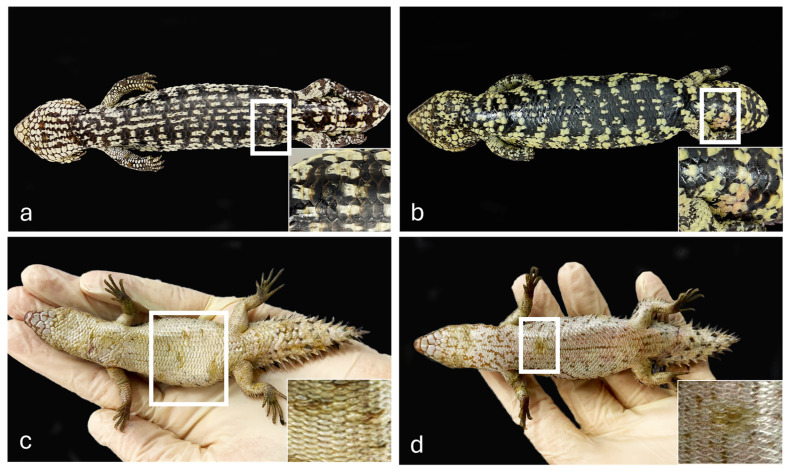
Gross skin lesions in Shingleback skinks from Queensland and Spiny-tailed skinks from Western Australia, Australia. Shingleback skinks. Cases 1–2. (**a**) Focal scale roughening on the caudal ventral body and focal reddened discoloration on the lateroventral aspect of the tail (**b**). Spiny-tailed skinks. Cases 3–4. Multifocal ventral yellow discoloration and scale roughening on the mid to caudal ventral body (**c**) and focally on the cranial ventral body (**d**).

Both shingleback skink specimens showed skin lesions on the ventral aspect of the body, ranging from scale roughening caudally (Figure 1a) to reddened scale discoloration on the tail (Figure 1b). Internally, the skinks presented with catarrhal enteritis with multiple intraluminal cestodes. The spiny-tailed skinks showed multifocal yellow discoloration and moderate roughening of the scales of the ventral aspect of the body, in the mid to caudal ventral body (Figure 1c) and the cranial ventral body (Figure 1d).

### 3.2. Histopathology

**Figure 2:**
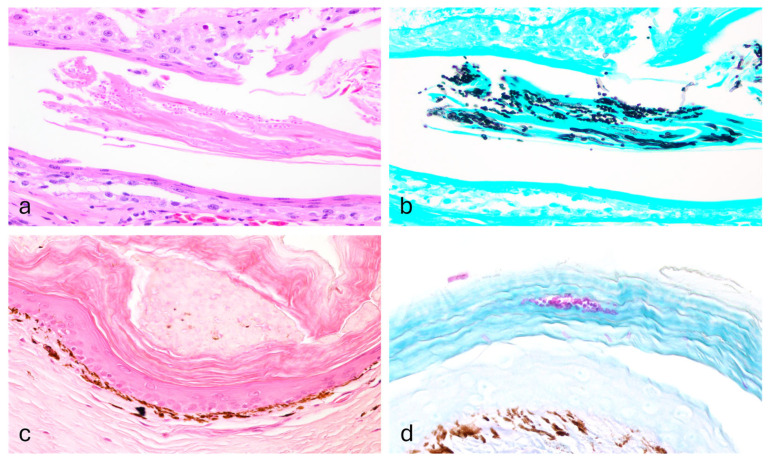
Histopathology of *Nannizziopsis pluriseptata* infection in the skin of a Shingleback skink and a Spiny-tailed skink. The epidermis shows mild orthokeratosis and within the stratum corneum there are multiple fungal structures (Case 1; (**a**), H&E, 40×), consistent with hyphae and arthroconidia (Case 1, (**b**); GMS, 40×). The epidermis shows moderate orthokeratosis (Case 3; (**c**), H&E, 40×) with multiple arthroconidia highlighted within the corneal layer (Case 3; (**d**), PAS, 40×).

One shingleback skink (Case 1) showed orthokeratotic and parakeratotic hyperkeratosis, with numerous intracorneal arthroconidia and hyphae, while both spiny-tailed skinks (Cases 3 and 4) showed parakeratosis, perivascular lymphoplasmacytic dermatitis with fungal structures (Figure 2). Granulomatous pneumonia with intralesional bacterial colonies was present as well. Enteritis was found in both shingleback skinks (Cases 1 and 2) along with intraluminal cestodes; embryonated cestode eggs were observed in only one case. Few intratubular crystals, compatible with calcium oxalate were found along with a mild focal granulomatous reaction in the kidney (Case 1).

### 3.3. Fungal Culture and Identification

18S sequencing of cultured isolates confirmed that *Nannizziopsis pluriseptata* was isolated from one of the Shingleback skinks (Case 1), while in the other specimen, the *Fusarium oxysporum* complex was recovered (Case 2). Fungal isolation was not performed on either of the samples from the spiny-tailed skinks.

### 3.4. PCR, Sequencing and Phylogeny

*Nannizziopsis* sp. was detected by PCR in a section of FFPE skin from one of the shingleback skinks (Case 1; Table 1). *Nannizziopsis* sp. was not detected by PCR in sections of fresh skin from both shingleback skinks (Cases 1 and 2) but was detected by the same method from both spiny-tailed skinks (Cases 3 and 4). After removal of primer sequences, the remaining 220-nucleotide segments of *Nannizziopsis* DNA were 99.1% (Case 1) and 99.5% (Cases 3 and 4) identical to a published sequence of *N. pluriseptata* (GenBank accession number: NR_111524.1). The sequences from Cases 1, 3, and 4 have been deposited into GenBank under the accession numbers PX672290 (Case 1) and PX672291 (Cases 3 and 4).

**Figure 3:**
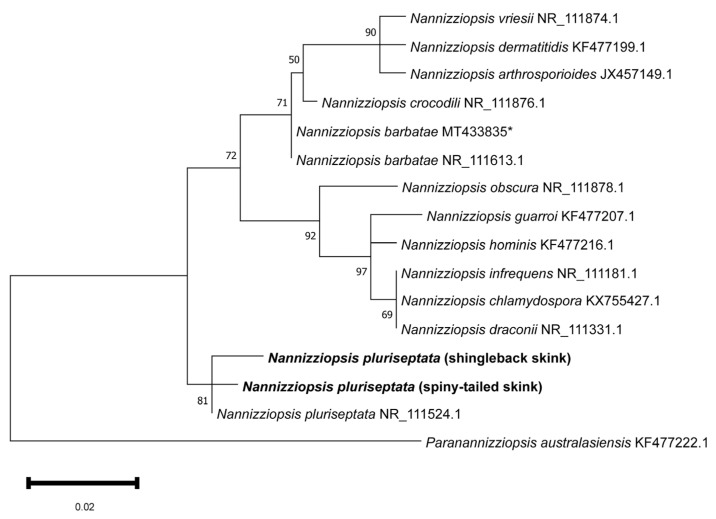
Maximum likelihood phylogenetic tree of a MUSCLE alignment of homologous partial *Nannizziopsis* nucleotide sequences of rRNA-ITS2. *Paranannizziopsis australesiensis* was used as an outgroup. Confidence of the tree topology is shown by maximum likelihood bootstrap values. Branch labels represent the fungal species and its GenBank accession number. The shingleback (Case 1) and spiny-tailed (Cases 3 and 4) *Nannizziopsis* sequences are in bold. * A sequence of *Nannizziopsis barbatae* from an Australian skink (*Tiliqua* spp.).

Phylogenetic analysis of the partial rRNA-ITS2 nucleotide sequences demonstrated that the shingleback and spiny-tailed skink *Nannizziopsis* sequences clustered tightly and exclusively with the *Nannizziopsis pluriseptata* reference sequence (NR_111524.1), forming a well-supported monophyletic clade (81% bootstrap) (Figure 3). Other species of *Nannizziopsis* grouped largely in accordance with established phylogenetic relationships and *Paranannizziopsis australasiensis* formed a well-supported outgroup [22].

## 4. Discussion

We report the identification of *N. pluriseptata* in two different reptile species derived from geographically distant regions in Australia, and the first report since the species was identified from the skin of a five-lined skink (*Plestiodon inexpectatus*) in North America [4].

*Nannizziopsis* spp. are a diverse group of fungi with unknown host specificity but are considered primary pathogens in reptiles [5,23]. It is not known how *N. pluriseptata* came to affect different reptile species from two geographically separated Australian states. It is possible that there could have been recent introductions of this fungus via the illegal pet trade due to the mobilization of animals and substrate, resulting in multi-location infection. Notably, little is known about the epidemiology of *N. pluriseptata* to support this possibility. It is also plausible that *N. pluriseptata* is an endemic, opportunistic fungus that only manifests disease when driven by stress, such as during capture, transportation, or captivity. To explore this idea further, active surveillance studies are needed, focused on sampling wild populations or archival museum specimens [24,25].

Diagnostically, mycotic culture and histological assessment are routinely carried out to identify fungal organisms. However, both have limitations because selective culture agars are often required to control contaminant fungal growth, and morphologically, it is challenging to characterize fungal species [26]. Thus, molecular methods of PCR and sequencing must be carried out to correctly identify species. In Case 2, *N. pluriseptata* was not detected by PCR; instead, *Fusarium oxysporum* was cultured from skin lesions. *Fusarium* spp. has been proven to induce severe systemic disease due to its angioinvasive tendency in immunosuppressed mammals and has been detected among other fungus species, from skin lesions in snakes [27,28]. It is unclear whether this finding corresponds to a skin contaminant or a true pathogenic infection.

Nannizziomycosis can range from mild to severe skin lesions, extending from crusts to ulcerations and necrosis, and affected individuals can present with lethargy and emaciation [9]. In the present study, *N. pluriseptata*-infected skinks showed discrete gross lesions. The epidermis is usually the most affected structure in *N. barbatae* infection—just like these *N. pluriseptata* cases—but since the skinks submitted for post-mortem examination were euthanized, disease progression could not be assessed.

Among the *Nannizziopsis* spp., *N. crocodili* has only been reported in crocodiles, *N. dermatitidis* infects lizard species and *N. hominis*, *N. infrequens*, and *N. obscura* have been recovered only from humans [7,29,30]. Recently *N. arthrosporioides* has been linked to clinical disease in an African side-neck turtle (*Pelomedusa subrufa*), a Central American boa (*Boa imperator*) and a group of ball pythons (*Python regius*), and humans [31,32,33]. *Nannizziopsis guarroi* was previously known to only infect lizards, but experimental infection in corn snakes (*Pantherophis guttatus*) has demonstrated that the natural host ranges of this fungus may be under-reported [34].

Nannizziomycosis has been associated with death in captive reptiles, but infections in wildlife are less frequently reported. Fungal pathogens could spillover over into wild populations reaching distinct geographic areas through specimen movement and fomites; impacting survival in immunologically naïve groups [35,36,37]. However, studies on an urban population of eastern water dragons (*Intellagama lesueurii*) with *N. barbatae* have demonstrated a variability in the severity of the infection among individuals, lack of mass mortality and prolonged survival times of up to several years with the presence of skin lesions, which might imply a potential carrier role in these reptiles [37,38].

All cases in this study were confiscated from illegal trade, presumably to supply the demand for exotic reptile pets overseas. Because of this, there is a lack of information on the provenance and previous handling. Therefore, the identification of *N. pluriseptata* could imply its presence in wild reptile populations, but certain aspects of its ecology are unknown. Most reports in the literature note the detection of *Nannizziopsis* spp. in cutaneous lesions in captive reptiles, such as an inland bearded dragon (*Pogona vitticeps*), a green iguana (*Iguana iguana*), a Cuban rock iguana (*Cyclura nubila*), geckos and a centralian blue-tongued skink (*Tiliqua multifasciata*) [9,39,40]. However, there are examples of infections in free-living reptiles and captive reptiles of wild origin [9,13]. Wildlife trade remains a concern for reptile populations where they are usually related to a specialized market for live specimens held in captivity. According to the IUCN (International Union for Conservation of Nature) Red List, cited by the UNODC (United Nations Office on Drugs and Crime), 46% of the seized reptile species worldwide are threatened or near threatened [41]. In Australia, in the threatened categories, 28 squamate species (3%) were considered vulnerable, 26 (2.7%) were endangered, 10 (1.1%) critically endangered, two species (*Lepidodactylus listeri* and *Cryptoblepharus egeriae*) were assessed as Extinct in the Wild and one species (*Emoia nativitatis*) was considered to have recently become extinct [42]; therefore, the potential introduction and spread of fungal disease into these populations raises an important concern for reptile conservation.

The understanding of fungal infections in reptiles continues to grow. Despite being first recognized decades ago, onygenalean fungal disease in reptiles has been detected in ethanol-preserved snake specimens in museums, dating the origins of this disease to even earlier times [24,25]. To this end, it is possible that earlier reports of fungal disease in Australian reptiles may have been misclassified. In a report from 1985, an eastern blue-tongued skink (*Tiliqua scincoides scincoides*) was described as having skin lesions on the limbs similar to those now known to be associated with infection by *Nannizziopsis* species. At the time, disease was attributed to *Trichophyton terrestre*, but the possibility of this simply being a surface contaminant was not excluded since *T. terrestre* is one of the *Trichophyton* species for which *Nannizziopsis* fungi have been mistaken [26,43]. More recently, severe dermal disease in a centralian blue-tongued skink (*Tiliqua multifasciata*) was confirmed as *N. barbatae* infection [9].

Transmission is achieved by direct contact with infected individuals, although fomites can represent an important source of infection [23]. For example, *N. guarroi* persists in solid and aqueous substrates for up to 14 days. Exposure to sodium hypochlorite (bleach) can disinfect culture plates with *N. guarroi* isolates; as well as benzalkonium chloride and polyhexanide disinfectant; however, surface disinfection may only be suitable for captive reptiles [44,45]. Spiny-tailed skinks in this study were held in poorly ventilated containers, one of them having a moist substrate which may have facilitated infection. Another factor affecting pathogen transmission within a population is the association between social interactions and skin lesions. In eastern water dragons infected with *N. barbatae*, it was observed that social interactions did not depend on the presence or absence of disease but rather on the severity of lesions [38].

Emerging fungal diseases have become a worldwide concern. The expansion of these organisms and their associated diseases is multifactorial and is determined by complex interactions between biotic and abiotic components. These include the evolutionary potential of the fungus, host susceptibility, the dispersal of the agent secondary to host species redistribution (from illegal wildlife trade), contamination of traded goods, or long-distance spore dispersal [2]. Additionally, anthropogenic factors like population growth, urbanization and globalization, along with a changing climate could further enhance fungal pathogen transmission [2,46].

## 5. Conclusions

We report novel cases of *Nannizziopsis pluriseptata* in two species of Australian skinks. *Nannizziopsis* infections in reptiles have been described mostly in captive specimens, and these studies have provided knowledge on their pathogenesis, phylogeny, ecology, and specific host–pathogen interaction. The ecological disruption caused by illegal wildlife trade and subsequent dissemination of pathogens is a concern for the health of reptiles, as more species will encounter pathogens that they have not co-evolved with, posing significant challenges to immunologically naïve populations. The virulence and host-specificity of newly described or emerging *Nannizziopsis* species is unknown, illustrating the need for further research to assess the impacts on reptile populations.

## Figures and Tables

**Table 1:** Tests results for Cases 1–4. Histological evidence of fungal structures was demonstrated in the skin of Cases 1, 3 and 4. *Nannizziopsis* was cultured in Case 1. *Nannizziopsis pluriseptata* was detected via PCR in Cases 1, 3 and 4 and results were confirmed by Sanger sequencing. FFPE = formalin-fixed paraffin-embedded.

Case No.	Host species	Histology	Fungal culture	*Nannizziopsis* PCR result
Case 1	Shingleback skink	Detected	*Nannizziopsis*	FFPE skin-Detected Fresh skin-Not detected
Case 2	Shingleback skink	Not detected	*Fusarium oxysporum*	Fresh skin-Not detected
Case 3	Spiny-tailed skink	Detected	Not performed	Skin swab-Detected
Case 4	Spiny-tailed skink	Detected	Not performed	Skin swab-Detected

## Data Availability

The original contributions presented in this study are included in the article. Further inquiries can be directed to the corresponding author.

## Abbreviations

The following abbreviations are used in this manuscript:

BLASTN	Basic Local Alignment Search Tool Nucleotide
CANV	*Chrysosporium* anamorph of *Nannizziopsis vriesii*
DCCEEW	Department of Climate Change, Energy, the Environment and Water
DETSI	Department of Environment, Tourism, Science and Innovation
DNA	Deoxyribonucleic acid
EPBC Act	Environment Protection and Biodiversity Conservation Act 1999
FFPE	Formalin-fixed paraffin-embedded
GMS	Grocott’s methenamine silver
H&E	Hematoxylin and eosin
IUCN	International Union for Conservation of Nature
ITS	Internal transcribed spacer
MUSCLE	Multiple sequence comparison by log-expectation
NCBI	National Center for Biotechnology Information
PAS	Periodic acid-Schiff
PCR	Polymerase chain reaction
PDA	Potato Dextrose Ager
rRNA	Ribosomal ribonucleic acid
SDA	Sabouraud Dextrose Agar
UNODC	United Nations Office on Drugs and Crime
